# Endoscopic iatrogenic esophageal perforation and management: a retrospective outcome analysis in the modern era

**DOI:** 10.1186/s12876-023-03004-x

**Published:** 2023-10-31

**Authors:** Eric M. Montminy, Blake Jones, J. Christie Heller, Augustin Attwell

**Affiliations:** 1 Division of Gastroenterology and Hepatology, University of Colorado Anschutz Medical Center, Aurora, CO 80045 USA; 2 Division of Gastroenterology and Hepatology, Denver Health Medical Center, Denver, CO 80204 USA; 3 Denver Health Medical Center, 601 Broadway, MC 4000, Denver, CO 80203 USA

**Keywords:** Iatrogenic esophageal perforation, Interventional endoscopy, Adverse events

## Abstract

**Introduction:**

Iatrogenic esophageal perforation (IEP) is a severe adverse event (AE) of upper endoscopy procedures (UEPs) associated with morbidity. Management has shifted from surgery to endotherapy with clip closure (CC), self-expanding metal stent (SEMS), and vacuum therapy (VT). Limited analyses measure outcomes during contemporary interventional endoscopy periods.

**Methods:**

IEPs associated with EGD, upper EUS, small bowel enteroscopy (SBE), and ERCP at a 3-hospital academic center from January 2011 to December 2023 were identified retrospectively from a centralized AE database. Additional information was obtained from medical records. Statistical analysis was performed using Microsoft Excel and STATA.

**Results:**

Thirty-two IEPs from 26 EGDs, 4 EUS, 1 SBE, and 1 ERCP were identified. IEPs occurred mostly after dilation (bougie *N* = 7; balloon, *N* = 5) or foreign body removal (*N* = 6). Most IEPs occurred in the lower esophagus (*N* = 10) or gastroesophageal junction (*N* = 8). Diagnosis was made at a median 2 h after the injury by endoscopy (*N* = 14), CT scan (*N* = 12), esophagram (*N* = 5), or x-ray (*N* = 1). Initial treatment included conservative therapy alone (*N* = 7), CC (*N* = 3), SEMS (*N* = 14), SEMS plus CC (*N* = 3), or surgery (*N* = 3). Eleven patients required additional treatment including repeat SEMS or adjustment (*N* = 4) or VT (*N* = 1). No surgical interventions were required after 2013. The median hospital stay was 3 days. Disposition included discharge to home (*N* = 25), long-term care facility (*N* = 2), 4 deaths (12.5% of IEPs), and 1 unknown.

**Conclusions:**

IEPs are rare and occur throughout the esophagus after any UEP. The majority are recognized immediately and managed with endotherapy, or rarely, surgery today. These characteristics likely explain the low mortality in this study.

**Supplementary Information:**

The online version contains supplementary material available at 10.1186/s12876-023-03004-x.

## Introduction

**Fig. 1:**
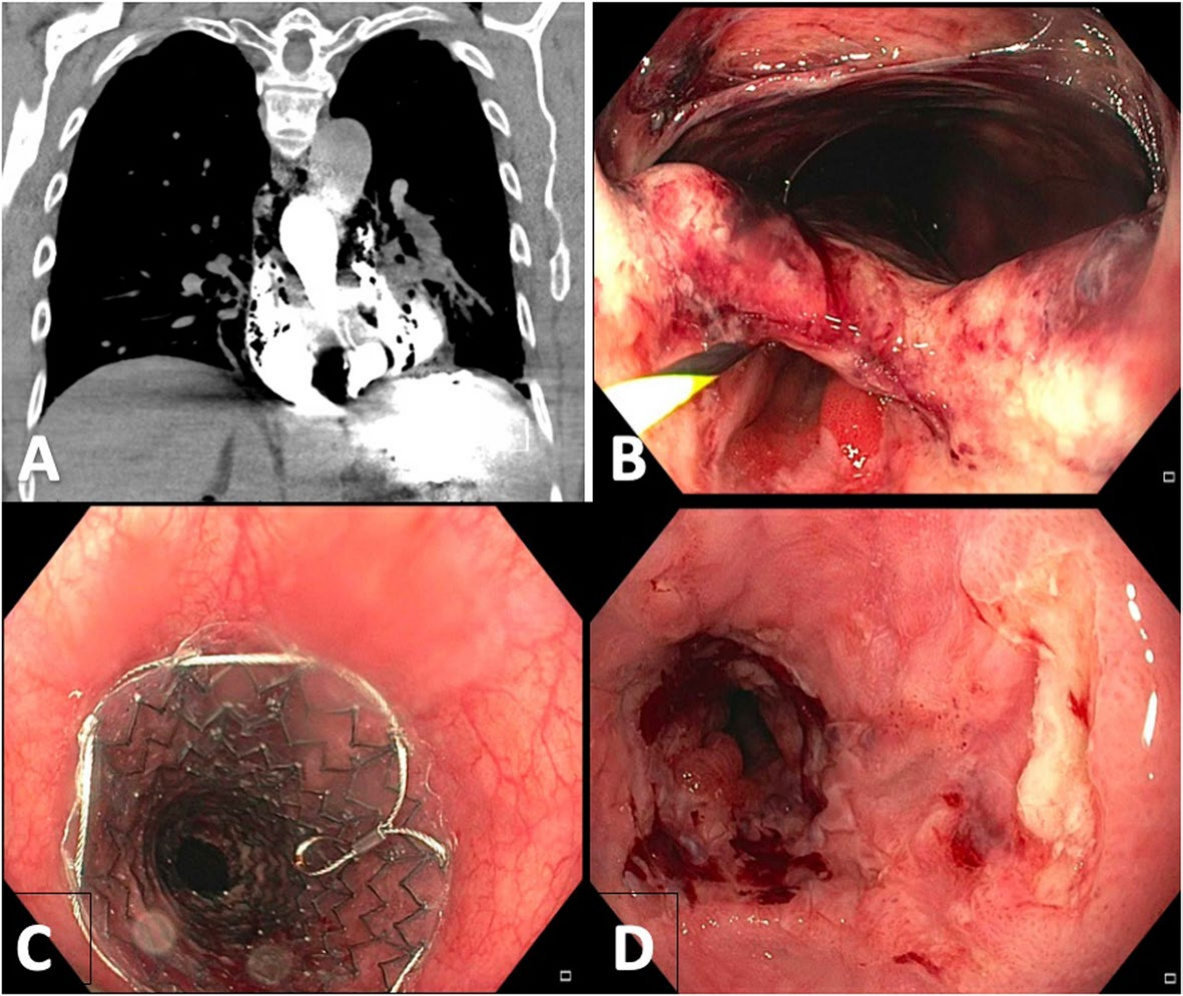
Iatrogenic esophageal perforation during an upper endoscopic procedure treated with a fully covered SEMS. **A** demonstrates the perforation in the esophagus represented on CT Chest with free air in the mediastinum. **B** demonstrates the endoscopic visualization of perforation with a guidewire in the esophageal lumen. **C** demonstrates placement of the fully covered SEMS. **D** demonstrates healing of the IEP after stent is removed

Upper endoscopic procedures (UEPs) and their interventions are safe, standard-of-care procedures used in the diagnosis and treatment of gastrointestinal diseases. Of the known adverse events (AEs), iatrogenic esophageal perforation (IEP) is considered one of the most severe because of the risk of exposure of the sterile mediastinum to bacteria, sepsis, and death. IEP is defined as a perforation of the esophagus byendoscopic instrumentation resulting in exposure of the esophageal flora to the mediastinum. The risk of death after esophagogastroduodenoscopy (EGD) is 0.01–0.05%, but IEP-specific morbidity and mortality rates after EGD and other UEPs are variable [1]. Endoscopic therapies including esophageal dilation, mucosal resection/submucosal dissection, and thermal therapy are associated with a small risk of IEP, and side-viewing procedures such as endoscopic ultrasound (EUS) or endoscopic retrograde cholangiopancreatography (ERCP) may also incur risk [2]. Historically, surgical repair was standard of care, but over the last 2 decades treatment has shifted towards endotherapy including covered self-expanding metal stent (SEMS) placement (Fig. 1), clip closure (CC), and endoluminal vacuum therapy (EVT).

Given the paucity of data on IEP outcomes since endotherapy became standard treatment, a fresh outcome analysis is warranted. Previous studies on IEP have been limited by its rarity and short follow-up even within high-volume centers. Additionally, prior studies have typically included all etiologies of perforation, which can result in generalization bias, since IEPs tend to be recognized earlier and may be managed differently than others. Herein, we analyze the outcomes of patients sustaining IEP after UEPs within a 3-hospital academic center over a 12-year period.

## Methods

Retrospectively identified IEPs were collected on patients from January 2011 to December 2023 at 3 hospitals: University of Colorado Hospital, Denver Health Medical Center, and the Rocky Mountain Regional Veterans Medical Center. UEPs included EGDs with various interventions, antegrade small bowel enteroscopy (SBE), EUS, and ERCP. Video capsule endoscopy (VCE) was excluded given its low risk and absence of associated IEPs during the study. Severe AE data were collected from a centralized reporting system, and IEPs were filtered from this list. The AE reporting system was based on paper reports/files from January 2011 to February 2020 and electronically using a centralized Research Electronic Data Capture (REDCap) system from March 2020 onward. All IEPs during EGD and EUS were esophageal interventions unless detailed (see Results). All IEPs related to ERCP or enteroscopy were intuitively not related to an esophageal intervention. Basic demographic and procedure data was obtained from the AE database, and the remaining information was obtained from the electronic medical record (MR) (EPIC, Racine, Wisconsin). Procedural data included patient age/gender, hospital, endoscopist, trainee involvement, significant past medical history, prior UEP data, type of UEP, intervention/s performed during the index procedure, exam indication, and IEP location. MR data also included IEP diagnostic testing and timing of diagnosis, length of hospital stay, treatment/s during the hospitalization, and final disposition/outcome. Delayed perforation was defined as a perforation being diagnosed after 48 h. All data points were verified for each IEP case and no cases were excluded.

This study was reviewed by the Colorado Medical Institutional Review Board and determined to be exempt from approval based on the absence of identifiable patient data or patient contact. The need for ethics review and patient consent were waived by the Colorado Medical Institutional Review Board. All procedures performed were in accordance with the ethical standards of the institutional research committee and with the 1964 Helsinki declaration and its later amendments or comparable ethical standards. All data is provided (See supplement).

### Statistical analysis

Statistical analysis was performed using Microsoft Excel 2019 (Microsoft Corporation, Redmond, WA, USA). Additional data analysis was performed using STATA version 13.0 (Statacorp, College Station, TX, USA). Descriptive statistics are reported as frequencies and percentages where appropriate.

## Results

From January 2011 through December 2022, a total of 98,455 EGDs, 456 SBEs, 15,295 EUS, and 20,237 ERCPs (total 134,443 UEPs) were performed at the 3 hospitals. The average age of IEP patients was 61 years-old. Of the 32 IEPs, 17 occurred in women and 15 in men. Half of the IEPs (*N* = 16) involved a gastroenterology fellow. Two of the 32 IEPs occurred in patients with a known diagnosis of eosinophilic esophagitis. Eleven of the 32 IEPs occurred in patients presenting with a complaint of dysphagia (of which 4 had a known prior benign stricture or prior radiation or surgery for an esophageal malignancy). Regarding hospital sites, 23 IEPs were at University of Colorado Hospital, 6 were at Denver Health Medical Center, and 3 were at the Rocky Mountain Regional Veterans Medical Center.

A total of 32 IEPs occurred during this period, yielding an overall IEP rate of 0.024% or one out of every 4,166 UEPs (Table 1). IEPs occurred most often during EGD (*N* = 26, 81%), typically after interventions including bougie dilation (*N* = 7, 22%), foreign body removal (*N* = 6, 19%), pneumatic dilation (*N* = 5, 16%), argon plasma coagulation (APC) (*N* = 1, 3%), endoscopic mucosal resection (EMR) (*N* = 2, 6%), or submucosal dissection (ESD) (*N* = 2, 6%) (Table 1). Four additional IEPs (13%) occurred during EUS echoendoscope passage, including 2 after fine-needle aspiration (FNA), one after combined EGD/EUS with extraction of a large duodenal adenoma through the upper esophageal sphincter (UES), and one during scope passage through a Zenker’s diverticulum (more details below). Four IEPs occurred during EGD or ERCP scope passage, including 2 during EGD for hematemesis and 1 during ERCP in a patient with distal esophageal stricture. One IEP occurred with SBE during passage of the device-assisted enteroscope (Spirus Medical, West Bridgewater, MA).

**Table 1:**
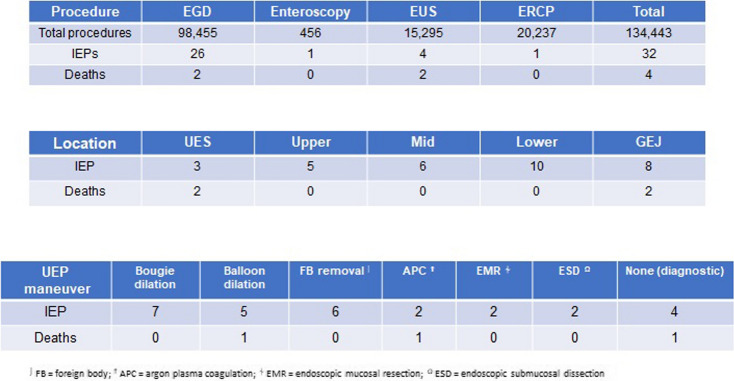
Upper endoscopy procedure totals with associated endoscopic intervention and IEP/death outcomes over January 2010 to December 2022

Specific procedural information regarding individual maneuvers is available from one of the 3 hospitals. At the University of Colorado Hospital (UCH), 87,268 total UEPs were performed during the study, including 3055 with balloon dilation, 2956 with bougie dilation, 1992 with foreign body extraction, 599 with EMR, 172 with ESD, 1428 with APC, and 77,066 without these interventions. The intervention-specific IEP rates for the various interventions are as follows: balloon dilation, 0.13% (4/3055); bougie dilation, 0.17% (5/2956); foreign body removal, 0.1% (2/1992); EMR, 0.33% (2/599); ESD, 1.2% (2/172); APC, 0.07% (1/1428). For UEPs without prior intervention, the IEP rate was 0.02% (16/77066).

Regarding location, 3 IEPs occurred at the UES, 5 in the upper esophagus, 6 in the middle esophagus, 10 in the lower esophagus, and 8 at the gastroesophageal junction (GEJ) (Table 1). The diagnosis of IEP was made at a median 2 h (0.5 -168 min) after the injury by endoscopy (*N* = 14, 44%), CT scan (*N* = 12, 38%), barium esophagram (*N* = 5, 16%), or plain x-ray (*N* = 1, 3%). The most common initial treatment of IEP was SEMS placement (*N* = 18, 56%) with or without CC at the same time (Table 2). Three patients (9%) underwent CC without SEMS. Conservative management alone – including intravenous antibiotics, nil-per-os, ± total parenteral nutrition—was administered to 7 patients. Surgery was performed or attempted as the initial treatment in 4 patients including open surgical repair of the defect with or without gastrostomy tube placement (GTP) (*N* = 3, 9%) and video-assisted thorascopic surgery (VATS) repair (*N* = 1).

**Table 2:**
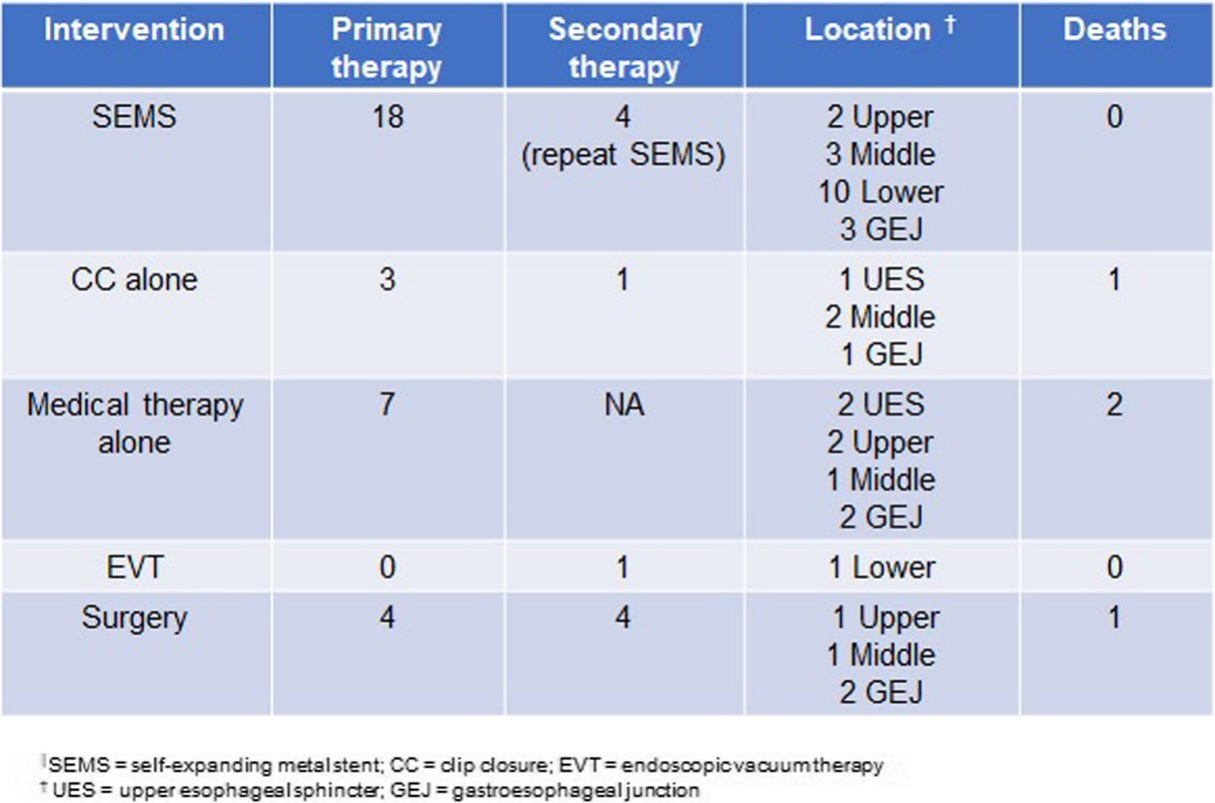
Initial endoscopic management and subsequent management of reported IEP over January 2010 to December 2022

No additional treatment was necessary beyond these measures in 21 patients. A repeat SEMS placement or adjustment was performed in 4 patients. One patient required EVT as rescue treatment after poor response to SEMS. One patient underwent repeat EGD with CC alone. Three patients underwent thoracoscopy or chest tube placement and 1 patient underwent surgical GTP for persistent symptoms or complications.

The median length of stay was 3 days after IEP with 25 patients being discharged home and 2 patients admitted to long-term care facilities. One patient left the hospital against medical advice. Four deaths (13%) occurred. Two deaths were associated with UES injury during attempted upper EUS. In one of these cases, EUS was performed for evaluation of a symptomatic duodenal polyp in an 87-year-old woman with cervical arthritis, and after EUS and polypectomy, peroral extraction of the polyp led to the IEP. Surgical repair was unsuccessful. In another case, EUS was attempted for evaluation of pancreatic and liver masses from suspected metastatic pancreatic adenocarcinoma, but passage of the linear echoendoscope resulted in IEP within a Zenker's diverticulum. Surgery was declined by the family. Another death occurred after APC of gastric vascular lesions in a 71-year-old male with multiple comorbidities including symptomatic anemia. The patient developed cardiac arrest en route to the operating room. Lastly, a frail 84-year-old male sustained a fatal IEP during 30-mm balloon dilation of the LES for treatment of symptomatic esophageal dysmotility. The patient declined surgery and transitioned to outpatient hospice care.

A total of 5 of the 32 IEPs were delayed perforations (Table 3). When comparing delayed and non-delayed perforations, delayed perforations resulted in longer median lengths of hospitalization (13 days vs 5 days). The majority of delayed perforation occurred at the GEJ (*N* = 3), and the majority of non-delayed perforations occurred in the lower esophagus (*N* = 10).

**Table 3:**
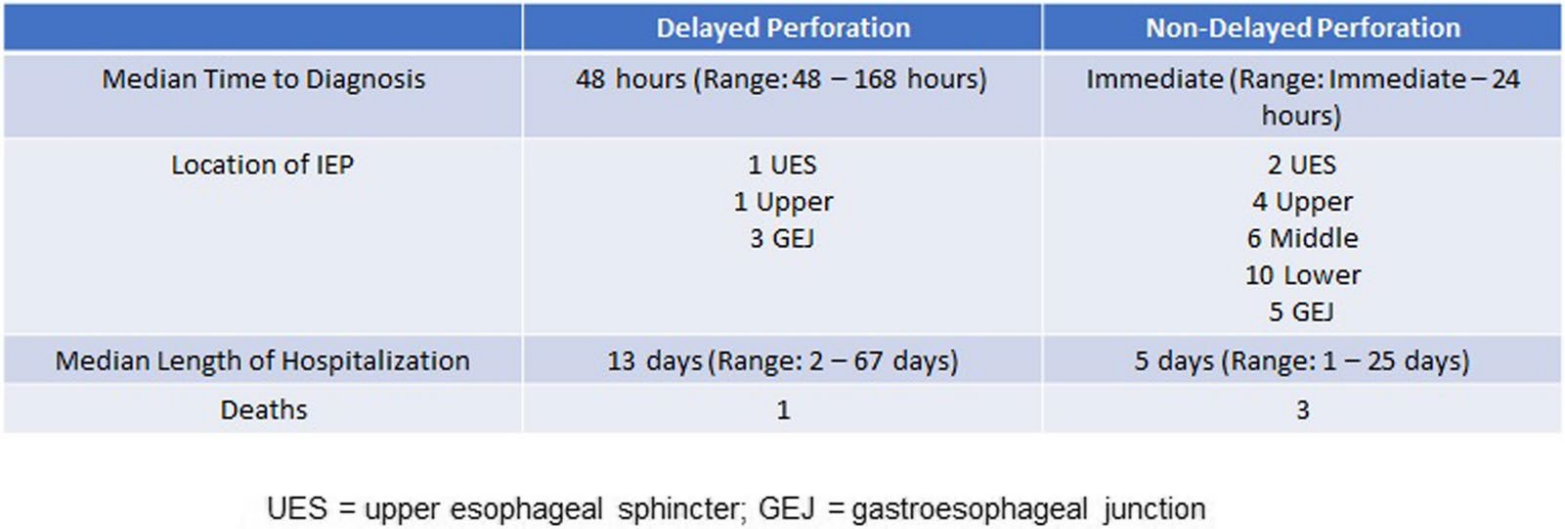
Comparison features of delayed and non-delayed IEPs

## Discussion

The current understanding of IEP outcomes remains limited and based largely on retrospective analyses prior to the development of advanced endotherapy. Iatrogenic injury during EGD or trans-esophageal echocardiography continue to be a common cause of perforations, as high as 46% [3]. Mortality rates from all types of esophageal perforations have varied considerably over the last 3 decades but have fallen to 15% or less with improvements in antibiotic therapy, diagnostic imaging, and surgical/endoscopic techniques [4]. The length of hospital stay for IEP is dependent on the type of intervention required, as those requiring surgical interventions (approximately 75% of interventions over 1995–2020) also require longer stays [5].

Our 12-year study shows that IEP is rare, occurring in approximately every 4000 UEPs, and associated with a < 13% mortality. IEP can be treated effectively with non-surgical methods, most commonly SEMS placement. Moreover, the median hospital stay was only 3 days and 25 (78%) patients were eventually discharged home.

IEP occurred most commonly after EGD with dilation or foreign body removal. Bougie and large-caliber pneumatic dilation appear to carry a higher risk than standard (20 mm or smaller) balloon dilation. Approximately two-thirds of IEPs required a single endoscopic intervention for definitive treatment. Most IEPs were recognized immediately or within 2 h of injury by endoscopic visualization or CT scan. Only 3 of the 32 perforations required surgery with none of these occurring after 2013, which further supports the effectiveness of contemporary endoscopic management. Of the 3 deaths related to IEP, 2 were associated with UES injury during EUS, suggesting that echoendoscope passage is more dangerous than forward-viewing scope passage, a trend demonstrated in previous studies [2].

Our study provides a granular evaluation of IEP outcomes particularly after endoscopic intervention. Prior studies of esophageal perforations, mostly from surgical literature, are limited by small and heterogenous patient populations and publication prior to the advent of modern endotherapy, which may result in worse outcomes and bias. The impact of etiology is crucial, because the clinical course shifts when the injury is recognized immediately during endoscopy (as was usually the case in our study) and prior to onset of mediastinitis. A prior study reported decreased mortality from iatrogenic IEP when diagnosed within 24 h compared to later [6]. Another large study that did not stratify outcomes by perforation etiology showed a mortality rate of 7.3% when SEMS was used for treatment [7]. This data echoes our low (0%) mortality rate after SEMS placement. Our data also demonstrates that SEMS is effective, with only 2 of the 17 IEPs requiring additional treatment. In prior studies it is unclear when and where the SEMS was placed relative to the IEP, so its benefit may have been underestimated and outcomes impacted accordingly. The higher mortality rates after SEMS in prior studies may also be attributed to selection of higher risk patients too ill for surgery [7].

Only 2 of the 32 IEPs occurred during diagnostic EGD, and 19 of the 26 patients with IEPs had prior EGDs. Hence, though rare, IEP during purely diagnostic EGD can and still does occur (2 of 98,455 EGDs) and a prior understanding of the patient’s upper GI anatomy evidently does not eliminate the risk. Our data also show that IEPs occur even throughout the esophagus, presumably as a result of diseases (particularly stenosis) in all segments. Interestingly, no IEPs related to ESD occurred in the last 10 years of the study. This may be related to a type 2 error and/or related to inexperience with technique as ESD is a newer endoscopic technique that has not been ubiquitously incorporated into most general gastroenterology and interventional endoscopy fellowships.

Of the 3 IEPs at the UES, 2 associated with EUS resulted in death. Perforations at the UES have limited options for endotherapy since the smaller space makes visualization and instrument passage difficult, and SEMS or EVT in this area may result in aspiration, airway compression, and/or patient intolerance. One of the patients with fatal UES injury after EUS was taken to the operating room but the only intervention feasible was drain placement, which failed to improve her condition. An increased mortality risk with UES perforation compared to other IEP locations has been suggested previously [8]. Increased mortality related to perforations in the intrathoracic esophagus compared to the lower esophagus has also been reported [6]. This trend may relate at least partially to the size and shape of the distal esophagus and surrounding structures relative to the cervical esophagus, as mentioned above. Additionally, perforations involving > 50% of the lumen or associated with frank mediastinitis may still require surgery for repair and/or drainage.

Another likely contributor to the low mortality rate and short hospital stays in our study was prompt recognition. IEPs were diagnosed during the procedure by endoscopy or later that day by contrast CT scan within a median 2 h. The increase in morbidity and mortality related to delays in diagnosis of perforation is a well-recognized phenomenon related to all areas of the GI tract [6]. Regarding early detection of IEPs, it should be stressed that any patient who develops chest pain, shortness of breath, or fever after EGD with therapeutic intervention should have a physical exam to evaluate for crepitus, a CBC with differential, chest x-ray, and/or CT Scan depending on the index of suspicion.

Finally, all 4 of the fatal IEPs occurred in patients with ASA class III or higher, and the chronic illnesses likely affected resilience to injury and subsequent intervention(s). The risk of mortality after IEP in ASA I-II patients is low, as shown in our study (0%, 18 patients) but needs to be confirmed in larger studies. Because IEP remains a devastating adverse event with significant mortality, patients with high ASA class (i.e. those unlikely to tolerate sepsis or thoracic surgery) should be treated with extra care. The use of fluoroscopy to monitor guidewire and bougie location during dilation should be utilized whenever possible to minimize false passages within the esophagus. A careful and thorough inspection of a stenosis or eosinophilic esophagitis after dilation and/or foreign body removal is also warranted.

The strengths of this study include a relatively large patient population (largest series of pure IEP available) from 3 different hospital systems and the complete inpatient data. Limitations include the retrospective design and the lack of any direct treatment comparisons. All UEPs were included, which dilutes the incidence of IEP but also represents a real-world clinical practice. Additionally, the patients were treated with state-of-the-art endoscopic and surgical techniques at an academic center, so the outcomes may not be applicable to other centers. The treatment of patients was determined on a case-by-case basis by individual physicians, so a standard protocol was neither established nor evaluated. Larger prospective studies of IEP outcomes in other centers will be needed to validate the treatment outcomes and determine which patients are best suited for specific endoscopic or surgical treatments based on injury size, location, age, and endoscopic or surgical expertise. These studies will require analysis of specific IEP sites and respective interventions in order to ultimately determine the best intervention for IEP by location. Finally, our study period mostly predated EVT. Future studies including this therapy may show further reductions in morbidity or mortality.

In conclusion, IEP is uncommon nowadays, but when recognized early, endoscopic therapy including CC or SEMS is effective and associated with a low risk of mortality or surgery. Early diagnosis and the location of most injuries in the middle or lower esophagus likely contribute to favorable outcomes. Large, multicenter, prospective studies are needed to validate the outcomes and match specific therapies to particular types of IEP.

### Supplementary Information

**Additional file 1.** RAW Data

## Data Availability

The data for this manuscript is provided as a supplemental file.
